# Effect of promoter, promoter mutation and enhancer on transgene expression mediated by episomal vectors in transfected HEK293, Chang liver and primary cells

**DOI:** 10.1080/21655979.2019.1684863

**Published:** 2019-10-31

**Authors:** Zhong-Jie Xu, Yan-Long Jia, Meng Wang, Dan-Dan Yi, Wei-Li Zhang, Xiao-Yin Wang, Jun-He Zhang

**Affiliations:** a Life Science and Technology, Xinxiang Medical University, Xinxiang, Henan, China; b Pharmacy collage, Xinxiang Medical University, Xinxiang, Henan, China; c International Joint Research Laboratory for Recombiant Pharmaceutical Protein Expression System of Henan, Xinxiang Medical University, Xinxiang, Henan, China; d Department of Biochemistry and Molecular Biology, Xinxiang Medical University, Xinxiang, Henan, China

**Keywords:** Gene expression, enhancer, episomal vectors, promoter, regulation of gene expression

## Abstract

The episomal vector cannot integrate into the host cell chromosome, which has no potential risk in gene therapy. However, the low level of transgene expression driven by episomal vectors needs to be solved. In this study, we investigated the effects of enhancers, promoters and promoter variants on transgene expression levels driven by episomal vectors in HEK293, Chang liver and primary cells. Results showed that all eight *cis*-acting elements used could increase transfection efficiency and transient *eGFP* expression in transfected HEK293 and Chang liver cells. In stably transfected mammalian cells, the elongation factor-1 alpha (EF-1α) promoter and mutant-404 showed high and stable transgene expression. The mechanisms might be related to the type and quantity of transcription factor regulatory elements. Moreover, quantitative reverse transcription polymerase chain reaction analysis showed that mRNA expression levels were not directly proportional to protein expression levels. Furthermore, the EF-1α promoter conferred high transgene expression levels in primary cells, and the plasmid was also present in the episomal state. Taken together, these results provided valuable information for improving transgene expression with episomal vectors in mammalian cells.

## Graphical Abstract

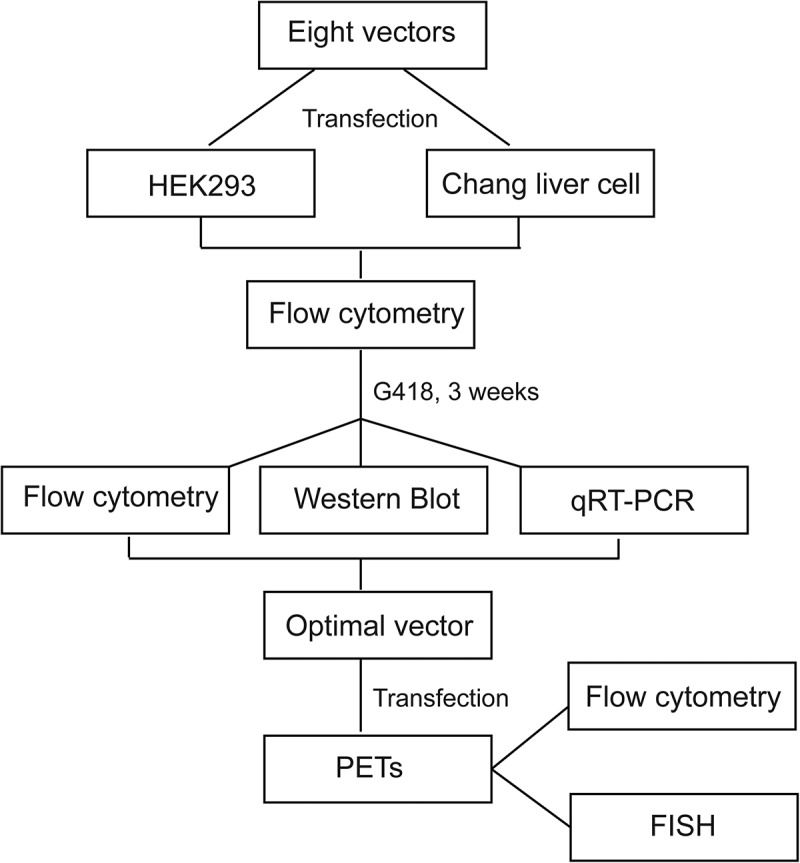

## Introduction

Gene therapy involves the delivery of a therapeutic gene into a patient’s cells as a drug to treat diseases. Vector systems play the key role in driving the expression of transgenes in gene therapy [1,2]. Episomal vectors can replicate in synchrony with each cell cycle division within the host genome for sustained, nonviral and nonintegrating transgene expression in vitro and in vivo [3–5], which does not cause insertional mutagenesis in gene therapy [6]. Therefore, the use of episomal vectors has obvious advantages in gene therapy. The first nonviral and episomal plasmid vector pEPI based on the matrix attachment region (MAR), which can replicate autonomously with low copy numbers in all cells tested, was developed by Piechaczek et al. [7]. However, the pEPI vector has several limitations, such as low copy number, unstable expression and low expression level [8,9].

Many studies have attempted to achieve high transgene expression levels via optimizing *cis-*acting elements in episomal vectors [10–12]. In our previous study, we constructed an episomal vector harboring a 387-bp DNA sequence containing a characteristic MAR motif driven by the cytomegalovirus (CMV) promoter; the construct was expressed in Chinese hamster ovary (CHO) cells in the episomal state [13]. However, the episomal vectors currently used for transgene expression often result in low levels of expression [14]. Therefore, the vector needs to be further optimized to achieve high expression level and stability.

Plasmid vectors contain some *cis*-acting elements, including promoters, enhancers, polyadenylation signals and other expression elements, which all affect transgene expression levels. Hagedorn et al. showed that the insulator sequence (cHS4) and a ubiquitous chromatin-opening element (UCOE) can improve expression and facilitate the establishment of a nonviral vector [15]. Benjamin et al. demonstrated that the CMV promoter mutants show a reduced propensity for productivity loss in CHO cells [16]. Although CMV is a strong promoter, some studies have shown that this promoter is intrinsically susceptible to transcriptional silencing associated with DNA methylation [17,18]. Alternatively, a variety of strong promoters, including human elongation factor-1 alpha (EF-1α) promoter and CAG promoter (a combination of the CMV immediate early enhancer and the chicken β-actin promoter), have been exploited to achieve the high-level expression of various genes [19,20].

In our previous study, we investigated enhancers, various promoters and promoter variants on transgene expression in CHO-K1 cells and found that the EF-1a promoter is a potent regulatory sequence for episomal vectors and maintains high transgene expression [21]. However, only the CHO-K1 cell line, which was unfit for gene therapy owing to its differences from human cells, was tested. In this study, we will optimize the episomal vectors with different *cis*-acting elements, including enhancer elements, EF-1α and CAG promoters and CMV promoter mutant, to explore their effects on transgene expression in humanized HEK293, Chang liver and primary cells and their molecular mechanisms.

## Materials and methods

### Vector construction

**Figure 1.**
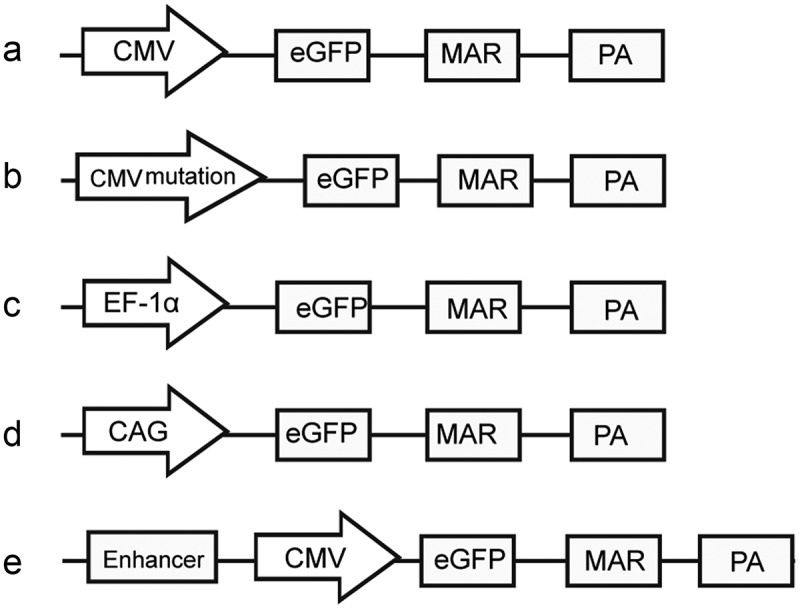
Schematic of expression vectors containing different elements. All vectors were derived from pEM (a). The DNA element comprising the characteristic MAR motif from the human IFN-β gene was cloned into the multiple cloning site (MCS) of pEGFP-C1, resulting in the pEM vector. (b) CMV promoter mutants: the cytosine at positions 404 bp and 542 bp in the CMV promoter was point-mutated to create two CMV mutant episomal vectors, which were named mutant-404 and mutant-542. (c) Vectors containing the EF-1α promoter were generated using the CMV promoter, which was excised and replaced with the EF-1α promoter. (d) Vector including the CAG promoter was generated by the CMV promoter, which was excised and replaced with the CAG promoter. (e) Three different enhancer elements were added upstream of the CMV promoters to create three vectors with enhancer, which were named as enhancer-NGE, enhancer-EEN and enhancer-NNG.CMV: human cytomegalovirus immediate early promoter; eGFP: enhanced green fluorescent protein gene; MAR: matrix attached region; PA: polyadenylation signals; NEO: neomycin phosphotransferase coding sequences.

The CMV promoter mutants (including cytosines at positions 404 and 542 point-mutated to guanosines) were artificially synthesized by Sangon Biotech Co., Ltd. (Shanghai, China). The synthesized sequences replaced the CMV promoter (i.e. the CMV promoter was excised and replaced with CMV promoter mutants) in the previously described pEM vector (Figure 1(a)) [21], and they were named as mutant-404 and mutant-542 (Figure 1(b)). By contrast, the EF-1α and CAG promoters were generated using polymerase chain reaction (PCR). To achieve directional cloning, we introduced the *Ase* I/*Nhe* I enzyme site at the 5´ ends of primers. The PCR program was as follows: 95°C pre-denaturation for 3 min, 94°C for 40 s, 56°C for 30 s, 72°C for 30 s, 30 cycles and a final step at 72°C for 3 min. The PCR products were recovered, and their sequences were confirmed, followed by digestion with the *Ase* I/*Nhe* I enzyme (TaKaRa Biotechnology Co. Ltd., Dalian, China). The products were then ligated into the pEM vector to produce vectors containing the EF-1α and CAG promoters (Figure 1(c,d)). Three different enhancer elements were also artificially synthesized by Sangon Biotech Co., Ltd. (Shanghai, China) and added upstream of the CMV promoters to produce three vectors containing different enhancers (Figure 1(e)). The three enhancers, which contained different combinations of nuclear factor (NF)-κB, E-box, GC-box and CCAAT-enhancer-binding protein alpha (C/EBPα) transcription factor regulatory elements (TFREs), were designed according to a previous report [22] and named as enhancer-NGE, enhancer-EEN and enhancer-NNG. The sequence of *cis*-acting elements used in this study is shown in Supplementary Figure S1.

### Cell culture and transfection

Human embryonic kidney cells (HEK293 cells; Institute of Laboratory Animal Sciences, Beijing, China) were grown in Dulbecco’s modified Eagle’s medium (Gibco, Carlsbad, CA, USA) containing 10% fetal bovine serum (FBS) and 1% penicillin–streptomycin. Human Chang liver cells (Institute of Laboratory Animal Sciences, Beijing, China) were maintained in Roswell Park Memorial Institute 1640 (RPMI 1640) medium (Invitrogen, MA, USA) supplemented with 10% FBS and 1% penicillin–streptomycin in a humidified incubator at 37°C with 5% CO_2_. Porcine fetal fibroblasts (PEFs) were isolated and cultured in DMEM containing 10% FBS, 1% glutamine (Gibco, MA, USA), 1% non-essential amino acids and 1% penicillin–streptomycin at 37°C and 5% CO_2_. After digestion, cells were centrifuged and seeded in 12-well plates. After reaching 80%–90% confluence, triplicate transfections were performed for each vector using Lipofectamine 2000 (Invitrogen) and PolyJet (SignaGen Laboratories, Rockville, MD, USA) according to the manufacturer’s instructions. Experiments were performed twice to ensure the reproducibility of the results (experiments 1 and 2 were performed using Lipofectamine 2000 and PolyJet transfection reagents, respectively). The cells in each well were transfected with different vectors using 2 µl Lipofectamine 2000 transfection reagent per 1 µg linearized plasmid DNA (plasmid DNA was digested with Nhe I) or 2.25 µl PolyJet transfection reagent per 0.75 µg linearized plasmid DNA. This study was conducted in accordance with the declaration of Helsinki and with approval from the Ethics Committee of Xinxiang Medical University.

### Transfection efficiency and transient expression

At 48 h post-transfection, the transfection efficiency and transient expression were analyzed by flow cytometry. HEK293, Chang liver and PEF cells were obtained and analyzed using a FACSCalibur cytometer (Becton Dickinson, Franklin Lakes, NJ, USA). A total of 100,000 fluorescent events were acquired using a 530/15 bandpass filter for the green fluorescent protein signal acquired with a fluorescence emission wavelength of 530 nm.

### Recombinant protein expression in stably transfected cells

Forty-eight hours after transfection, stably transfected cells were selected in the medium containing 800 µg/ml of Geneticin (G418, Invitrogen). Non-transfected cells died after 7–10 days of selection, and stably transfected pools were obtained after 3 weeks. The cells were further cultured in medium supplemented with 400 µg/ml G418 for 20 generations, and the eGFP stability expression was analyzed by flow cytometry. All transfections were carried out in triplicate.

### Western blot

Western blot was used to further analyze eGFP protein expression in stably transfected cells. A total of 5 × 10^6^ cells were collected and lysed with RIPA Lysis buffer (Beyotime, Shanghai, China). Approximately 10 µl of cell lysate was electrophoresed on a 15% sodium dodecyl sulfate-polyacrylamide gel electrophoresis (SDS-PAGE) gel. Subsequently, the proteins were transferred to a polyvinylidene fluoride membrane (Bio-Rad, USA) and reacted with 1:5000 diluted eGFP antibody (Beyotime, Shanghai, China) at 4°C overnight and then with goat anti-rabbit IgG antibody conjugated with horseradish peroxidase. After washing with PBS for 5 min, the protein bands were visualized using an enhanced chemiluminescence substrate kit (Amersham, GE Healthcare, Chicago, IL, USA). The relative expression values of protein were quantitatively determined using ImageJ software (version 1.41, National Institutes of Health, USA).

### qRT-PCR analysis

eGFP transgene expression at the mRNA level was detected in stably transfected cells and analyzed by quantitative reverse transcription PCR (qRT-PCR). From 5 × 10^6^ stably transfected cells, the total RNAs were isolated using TaKaRa RNAiso Reagent according to the manufacturer’s instructions (TaKaRa Company). RNA was converted to cDNA using a high-capacity cDNA Reverse Transcription Kit (Applied Biosystems, UK). qRT-PCR was performed on a Light Cycler 480 system (Roche) using the Roche LightCycler® 480 SYBR Green Master Mix. The *eGFP* primers were as follows: 5'-CTACGTCCAGGAGCGCACCATCT-3' (forward), 5'-GTTCTTCTGCTTGTCGGCCATGATAT-3' (reverse). GAPDH was used as an internal reference, and the primer sequences were as follows: 5'-CGACCCCTTCATTGACCTC-3' (forward), 5'-CTCCACGACATACTCAGCACC-3' (reverse). The qPCR procedure consisted of 40 cycles using the manufacturer’s recommended parameters. Relative eGFP mRNA was calculated using the 2^−∆∆^ Ct method. All experiments were repeated three times.

### Analysis of the status of vectors

To verify the status of vectors within the selected stable PEF cells, FISH was performed as described previously at 20 generation post-transfection [13]. *eGFP* probe was labeled using a digoxigenin-nick translation kit (Roche, Mannheim, Germany). The samples were counterstained with 1 μg/mL of 4',6'-diamidino-2-phenylindole and further analyzed using a Leica DMRB fluorescence microscope (Leica Microsystems, Wetzlar, Germany). Approximately 10 fields were observed.

### Bioinformatics analysis

TFREs were identified using the MatInspector software (http://www.genomatix.de/products/index.html) [23].

### Statistical analysis

All experimental data were analyzed using SPSS 18.0 software (SPSS Inc., Chicago, IL, USA). Data were reported as means ± standard deviations. A post-analysis of variance multiple comparison procedure was further performed to assess pairwise differences in expression confirmed by analysis of variance. Results with *P* values less than 0.05 were considered statistically significant.

## Results

### Transfection efficiency in HEK293 and Chang liver cells

**Figure 2.**
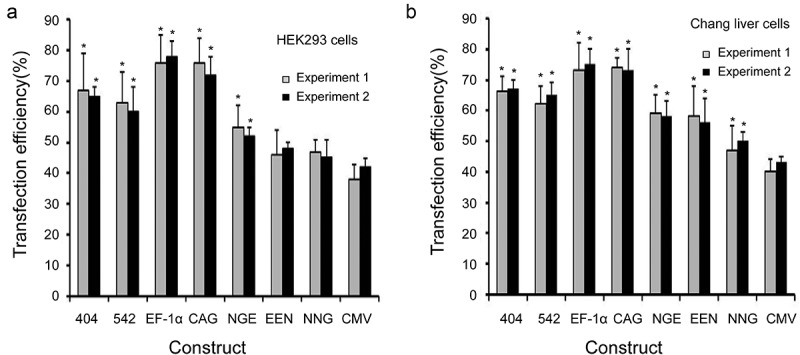
Transfection efficiency in transfected HEK 293 and Chang liver cells. The eight vectors were transfected into HEK 293 and Chang liver cells using Lipofectamine® 2000 and PolyJet transfection reagents according to the manufacturer’s instructions. The transfection efficiencies were determined by flow cytometry at 48 h after transfection. Three independent experiments were performed in this study. Gray and black bars represent the results from experiments 1 and 2, respectively. Experiment 1 was performed using Lipofectamine 2000 transfection reagent, and experiment 2 was performed using the PolyJet transfection reagent. Standard error of the mean (SEM) is indicated. * indicates that the transfection efficiency from different vectors was significantly higher than that from vectors containing the CMV promoter (Student’s t-test, *P* < 0.05).

We first evaluated the transfection efficiency of different enhancers, promoters and promoter variants in HEK293 and Chang liver cells. The transfection efficiency was the highest for plasmids containing the EF-1α promoter, followed by the CAG promoter, mutant-404, mutant-542, enhancer-NGE, enhancer-EEN, enhancer-NNG and CMV promoter (Figure 2). The transfection efficiencies of the plasmids under the EF-1α and CAG promoters, mutant-404, mutant-542 and enhancer-NGE were significantly higher than those under the CMV promoter in HEK293 cells (*P* < 0.05, Figure 2(a)). By contrast, in Chang liver cells, the transfection efficiencies under all elements used in this study were significantly higher than those under the CMV promoter (*P* < 0.05, Figure 2(b)). Moreover, no difference was observed between experiments 1 and 2, suggesting that the transfection efficiency was not affected by the transfection reagent.

### Transient expression in HEK293 and Chang liver cells

**Figure 3.**
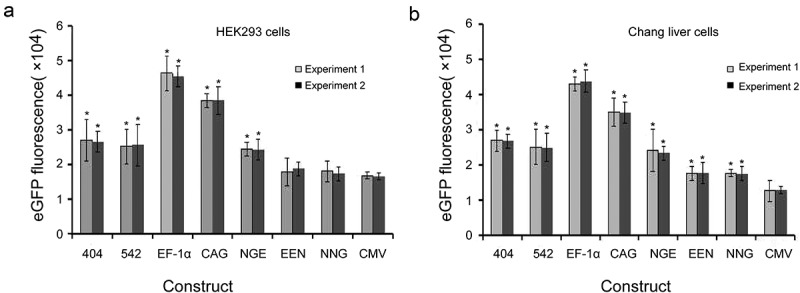
Transient expression of recombinant protein in transfected HEK293 and Chang liver cells. eGFP expression was determined by flow cytometry at 48 h after transfection. Three independent experiments were performed in this study. Gray and black bars represent the results from experiments 1 and 2, respectively. Each point represents the average and standard deviation of various vectors. * indicates that the eGFP expression levels from different vectors were significantly higher than those from vectors containing the CMV promoter (Student’s t-test, *P* < 0.05).

We evaluated the different elements individually to determine their abilities to enhance transient gene expression levels in HEK293 and Chang liver cells using eGFP as the reporter gene. The EF-1α promoter exhibited substantial enhancing effects in HEK293 and Chang liver cells, and transient eGFP levels were increased by approximately threefold. For the other *cis*-acting elements, the enhancing effects were observed in the following order: CAG promoter > mutant-404 > mutant-542 > enhancer-NGE > enhancer-EEN > enhancer-NNG > CMV promoter. Similarly, transient expressions of the eGFP gene under the EF-1α and CAG promoters, mutant-404, mutant- 542 and enhancer-NGE were also significantly higher than those under the CMV promoter in HEK293 cells (*P* < 0.05, Figure 3(a)). All elements used in this study induced significantly higher eGFP expression compared with that under the CMV promoter in Chang liver cells (*P* < 0.05, Figure 3(b)). When the *eGFP* expression level from plasmid pEM containing the CMV promoter was set to 1.0, the expression levels in HEK293 cells transfected with plasmid containing the mutant-404, mutant-542, EF-1α promoter, CAG promoter, enhancer-NGE, enhancer-EEN and enhancer-NNG were 1.61 ± 0.22, 1.55 ± 0.13, 2.76 ± 0.12, 2.33 ± 0.25, 1.51 ± 0.16, 1.18 ± 0.24 and 1.05 ± 0.11, respectively, and those in Chang liver cells were 2.08 ± 0.45, 1.97 ± 0.23, 3.40 ± 0.35, 2.71 ± 0.21, 1.78 ± 0.11, 1.37 ± 0.17 and 1.36 ± 0.13, respectively. Moreover, the eGFP transient expression showed no difference between experiments 1 and 2, suggesting that eGFP transient expression was not affected by the transfection reagent.

**Figure 4.**
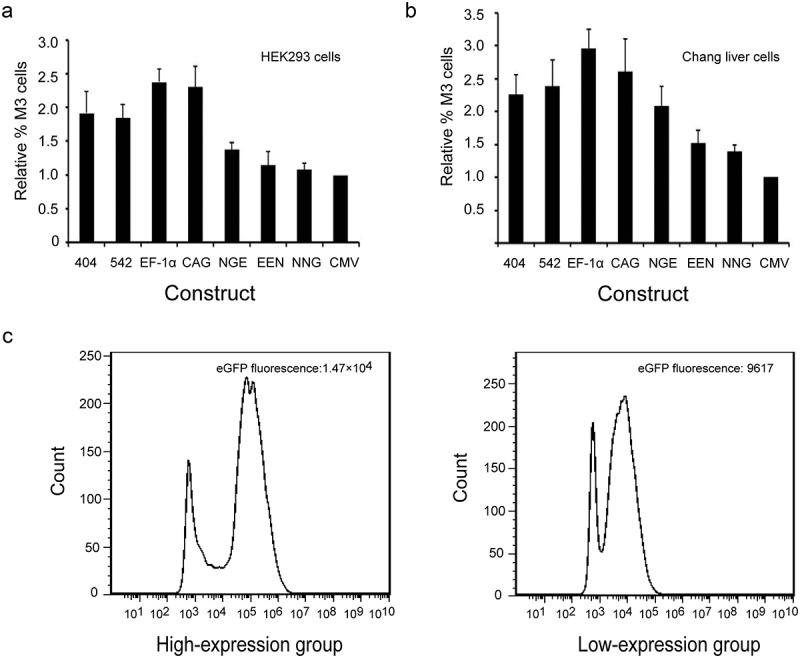
(a, b) Relative %M3 cells in transfected HEK293 and Chang liver cells. (c) eGFP expression levels in high- and low-expression groups with the EF-1α promoter.

To further evaluate the effects of different elements on *eGFP* transgene expression levels in HEK293 and Chang liver cells, the percentage of high producers (% M3, fluorescence values greater than 10^4^) was calculated. We analyzed the total population of eGFP-expressing cells and found that the EF-1α promoters resulted in the highest percentages of highly expressing cells (% M3) in transfected HEK293 and Chang liver cells (Figure 4(a,b)). Next, the cells were divided into high-expression (fluorescence values greater than >10^4^) and low-expression groups (fluorescence values smaller than <10^2^) by flow sorting in the plasmid with EF-1α promoters, and eGFP expression was detected by flow cytometry. The results showed that eGFP expression levels in the high-expression group was significantly higher than those in the low-expression group in HEK293 and Chang liver cells (*P* < 0.05). The representative results are shown in Figure 4(c).

### Stable expression in transfected HEK293 and Chang liver cells

**Figure 5.**
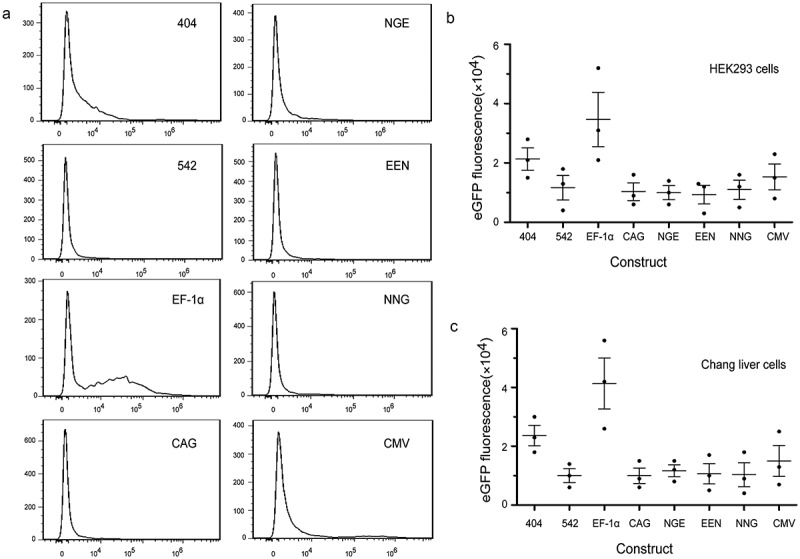
Expression stability of recombinant protein in transfected pools. At 48 h post-transfection, cells were subjected to G418 selection for establishing stable transfectants, and the eGFP expression levels were measured using flow cytometry. (a) Expression stability of recombinant protein in transfected pools was tested using a FACS Calibur instrument. (b, c) Analysis of eGFP expression level in HEK293 and Chang liver cells. Three independent experiments were performed in this study. The standard error of the mean (SEM) is indicated.

Forty-eight hours after transfection, the HEK293 and Chang liver cells were subjected to drug selection to establish stable transfectants. We cultured three colonies of stably transfected cells with drug agents for 20 generations, and eGFP expression was measured. The EF-1α promoter had the most stable expression, followed by mutant-404. The other elements showed less stable expression compared with the CMV promoter (one example is shown in Figure 5(a)). The eGFP expression levels under the EF-1α promoter in HEK293 and Chang liver cells were 3.47 ± 0.62 and 4.13 ± 0.54, respectively, which were higher than those under the CMV promoter (1.53 ± 0.27 and 1.50 ± 0.31, Figure 5(b,c)). Moreover, the eGFP expression levels with mutant-404 were also higher than those of the CMV promoter in HEK293 and Chang liver cells (2.03 ± 0.22 vs. 1.53 ± 0.27, 2.37 ± 0.40 vs. 1.50 ± 0.31; Figure 5(b,c)). These results suggested that the EF-1α promoter and mutant-404 played a role in strengthening and maintaining eGFP expression and that the EF-1α promoter was the most effective in maintaining recombinant protein expression stability.

### Analysis of eGFP protein expression

**Figure 6.**
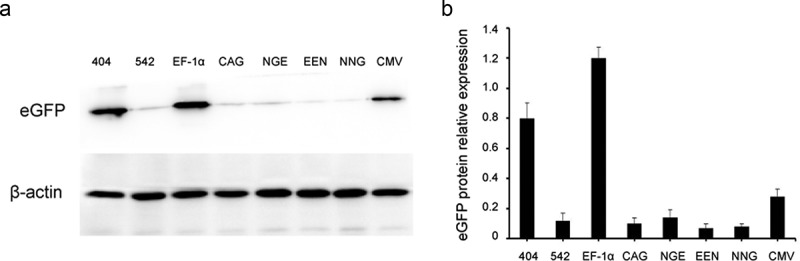
Analysis of eGFP expression using Western blot. Western blot analysis was used to detect eGFP expression levels in stably transfected HEK293 cells. The relative expression values of protein were quantitatively determined using ImageJ software.

To further investigate the effects of different *cis*-acting elements on recombinant protein expression, stably transfected cells were collected, and eGFP was detected by Western blot analysis. The results indicated that the eGFP expression levels by Western blot assay were consistent with those of flow cytometry. The EF-1α promoter had the highest eGFP expression, followed by mutant-404 and CMV promoter. The eGFP expression levels with EF-1α promoter and mutant-404 were higher than those of the CMV promoter in HEK293 and Chang liver cells. The representative results are shown in Figure 6.

### mRNA expression in transfected HEK293 and Chang liver cells

**Figure 7.**
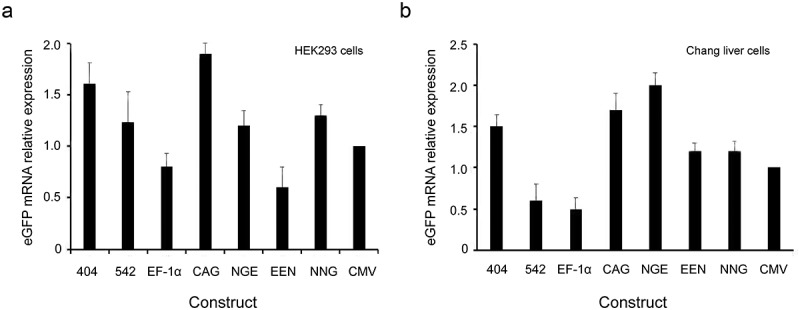
eGFP expression at the mRNA level. Stably transfected pools were generated by transfection of HEK293 and Chang liver cells with various vectors. The eGFP mRNA expression levels were measured by qRT-PCR. The error bars represent the standard deviation of three independent measurements.

We further analyzed the mRNA expression levels of stably transfected cells. The results showed that the mRNA expression was the highest in cells transfected with the CAG promoter, followed by cells transfected with mutant-404. The mRNA expression levels were not directly proportional to protein expression levels, suggesting that the regulatory mechanism after transcription may influence transgene expression (Figure 7).

### Transient and stability expression in PEF cell

**Figure 8.**
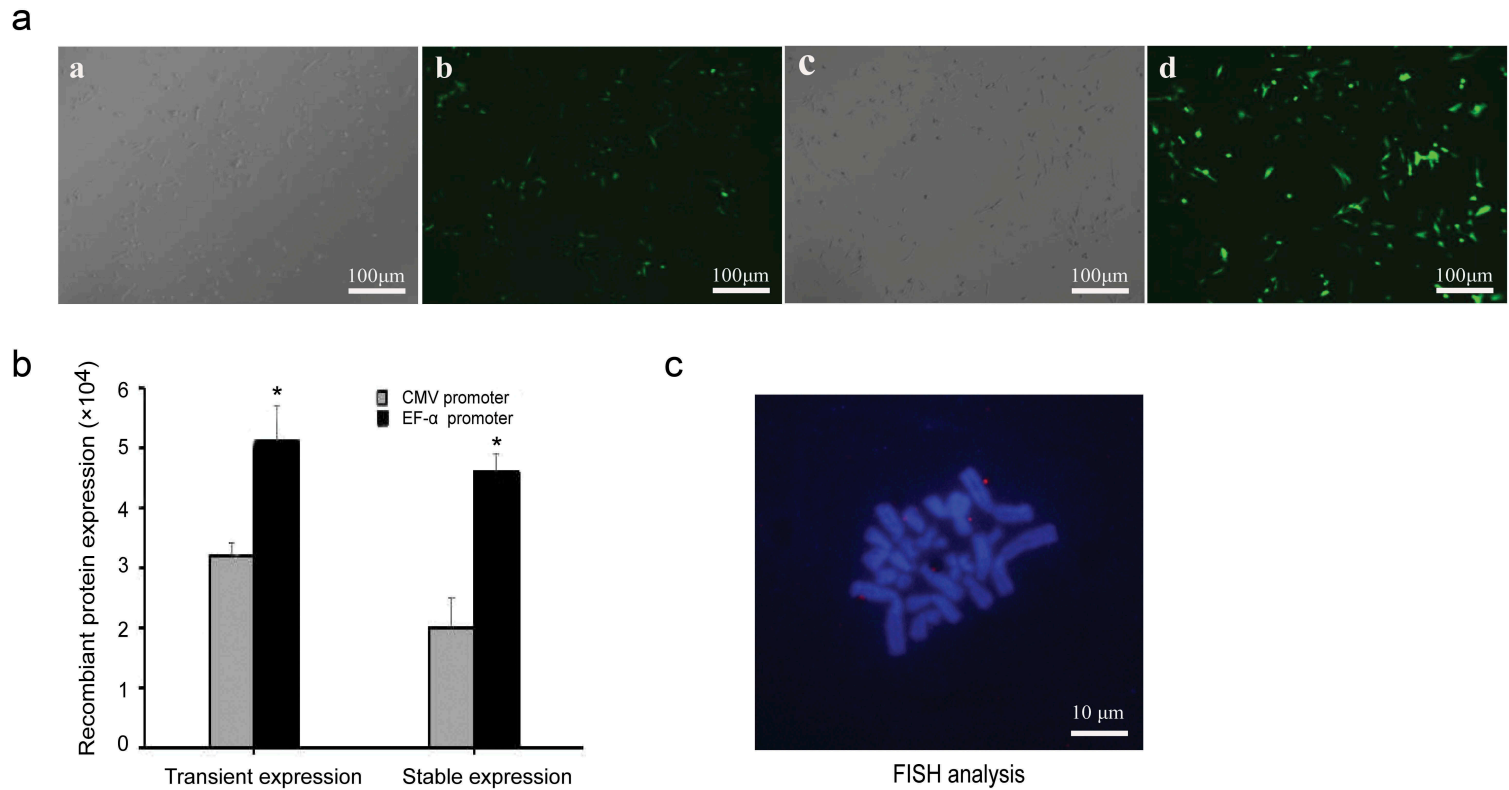
Recombinant protein expression and status of vectors in PEF cells. (a) eGFP expressions were observed under an epifluorescence microscope after 48 h of transfection. Cells transfected with plasmid containing the CMV promoter under white light (a) and fluorescence (b). Cells transfected with plasmid containing the EF-1α promoter under white light (c) and fluorescence (d). (b) eGFP expressions were determined by flow cytometry after 48 h transfection and at generation 20. Standard error of the mean (SEM) is indicated. * indicates that transgene expression with EF-1α promoter was significantly higher than that from vectors containing the CMV promoter (Student’s t-test, *P* < 0.05). (c) PEF cells transfected with plasmid containing EF-1α promoter at generation 20 were analyzed by FISH to assess whether the vectors were present as integrated copies. The episome (red) was visualized by eGFP FISH (Blue: metaphase chromosomes; red: vectors).

Given that the EF-1α promoter showed the best performance in terms of yielding both high expression levels and transfection efficiency, we chose the EF-1α promoter vector for transfection of primary cells. Forty-eight hours after transfection, eGFP gene expression was observed using an OLYMPUS IX71 fluorescence microscope, and the expression of eGFP was measured by flow cytometry at 48 h and 20 generations after transfection. In PEF cells, the EF-1α promoter produced relatively higher eGFP expression compared with the CMV promoter (Figure 8(a)). Flow cytometry showed that the eGFP expression levels with EF-1α and CMV promoters were 5.12 ± 0.53 and 3.26 ± 0.22 in transient expression and 4.67 ± 0.3 and 2.05 ± 0.6 in stability expression, respectively (Figure 8(b)). The EF-1α promoter increased the eGFP transgene expression by 1.6-fold and 2.3-fold than the CMV promoter in transient and stable cell pools, respectively.

### FISH analysis

FISH analysis was performed on PEF cells at generation 20, and 10 metaphase spreads were analyzed. FISH results revealed that the observed mitotic stability of the vector containing the EF-1α promoter was a result of the vector existing episomally on metaphase chromosomes in PEF cells. The representative results are shown in Figure 8(c).

### Analysis of the TFREs

Promoter activity is related to the transcription factor-binding sites and TFREs [24,25]. The distributions of seven TFREs (NF-κB, E-box, GC-box, C/EBPα, E4F1 and CRE) were assessed for the EF-1α, CAG and CMV promoters and enhancer-NGE, enhancer-EEN and enhancer-NNG. The NF-κB, E-box, C/EBPα, E4F1 and CRE TFREs were abundant in the enhancer-NGE, enhancer-EEN and enhancer-NNG (Table 1). The EF-1α promoter containing NF-κB had lower E4F1and CRE TFREs (Table 1). These findings led us to conclude that NF-κB, E-box, E4F1 and CRE TFREs relate to promoter activity the most.

**Table 1.** Locations of various transcription factor binding motifs within the promoters and enhancers.

Promoter/Enhancer	NFkB	E-box	C/EBPα	GC-box	E4F1	CRE
CMV	4	0	2	0	9	21
EF-1α	5	0	3	0	0	9
CAG	5	0	0	6	7	12
Enhancer-NGE+CMV	9	5	2	0	9	21
Enhancer-EEN+CMV	11	5	3	1	9	21
Enhancer-NNG+CMV	11	3	4	1	9	21

## Discussion

An ideal episomal vector for human gene therapy must provide high levels of transgene expression. In this work, we evaluated the effects of different *cis*-acting elements on transgene expression levels in HEK293, Chang liver, and primary cells (PEF cells). We demonstrated that enhancer elements, mutant CMV and CAG and EF-1α promoters increased transfection efficiency and recombinant protein transient expression. Moreover, the mutant-404 and EF-1α promoters increased recombinant protein stable expression in HEK293 and Chang liver cells. In PEF cells, the EF-1α promoter produced relatively higher eGFP expression compared with the CMV promoter, and FISH analyses indicated that the vector can replicate episomally with cell division.

Previous studies indicated that the endogenous mammalian promoters can provide higher expression than viral promoters [26,27]. The EF-1α promoter has been shown to be one of the strongest promoters in various cell lines [28,29]. Indeed, the EF-1α promoter is often active in cells where viral promoters fail to drive downstream gene expression and are gradually silenced [30,31]. Some studies have shown that promoters of endogenous mammalian genes, such as EF-1α, can be more resistant to silencing than viral promoters [31]. The CAG promoter, a combination of the CMV immediate early enhancer and the chicken β-actin promoter, has frequently been used to drive high-level gene expression in mammalian cells [32–34]. Moreover, the reported EF-1α and CAG promoter are mainly used for viral or integration vectors but not for episomal vectors. In this study, we found that the EF-1α promoter yielded the highest expression level in episomal vectors in transfected HEK293 and Chang liver cells, and CAG yielded high transgene levels in transient expression, which is consistent with a previous report [35]. Thus, these promoters may have an application potential in the design of episomal vectors for gene therapy use.

The CMV promoter is one of the strongest promoters in mammalian cells. However, the expression levels of genes from episomal vectors driven by the CMV promoter are often low [36]. As previously reported, the CMV promoter is prone to transcriptional silencing due to DNA methylation [37,38]. Mammalian DNA is predominantly methylated at cytosine bases that are part of CpG dinucleotides [16]. In the CMV promoter, the cytosines at positions 404 and 542 are frequently methylated [16]. To investigate whether the deletion of CpG sites can enhance CMV-driven gene expression in episomal vectors, we performed point mutations of C to G at positions 404 and 542 and studied the effects of these mutations on gene expression levels in transfected HEK293 and Chang liver cells. The results showed that the mutation of the CMV promoter increased the recombinant protein transient expression, which was consistent with the results of Benjamin et al. [16], and mutant-404 increased stable expression. Therefore, we believe that the point-mutated CMV promoter will be of great interest to scientists with broad research interests.

To further enhance the functions of promoters, various enhancer elements have been added upstream of the promoters [39]. The enhancers are *cis*-acting elements that can increase transcription levels. According to a previous study [22], we synthesized three different enhancer elements, including different combinations of NF-κB, E-box, GC-box and C/EBPα, and cloned these elements upstream of the CMV promoters. The results showed that all enhancers used in this study could increase the recombinant protein transient expression in transfected Chang liver cells. However, only enhancer-NGE could increase recombinant protein transient expression in HEK293 cells, and enhancer-EEN and enhancer-NNG did not promote any transcriptional enhancing activity. These results may be related to the enhancer specificity in different cell lines, but all enhancers could not maintain the long-term transgene expression.

Western blot analysis also confirmed that the EF-1α promoter and mutant-404 could increase stable recombinant protein expression in transfected Chang liver and HEK-293 cells, and no significant enhancement was observed in those with enhancers.

Research shows that the most active promoters contain relatively high numbers of NF-κB and E-box and a correspondingly low number of GC-box and C/EBPα blocks [22]. The distributions of positive (NF-κB, E-box), neutral (GC-box, C/EBPα) and negative (E4F1, CRE) TFREs were assessed for the EF-1α, CAG and CMV promoters and enhancers. The NF-κB and E-box TFREs were abundant in the enhancer-NGE, enhancer-EEN and enhancer-NNG, but the E4F1 and CRE TFREs were also abundant (Table 1). A high number of positive sites are apparently counteracted by high numbers of negative sites to produce relatively weak promoters. The EF-1α promoter had lower E4F1 and CRE TFREs, which may be the reason why the *EF-1α* promoter showed the most universal and highest enhancement of gene expression. These findings led us to conclude that the type and quantity of TFREs may contribute to promoter activity the most. This may be the reason for the different transgene expressions of different promoters and enhancers. However, the translation-promoting effects of enhancers may rely to some extent on elements associated with the host cell line, and they can be used according to different host cell lines in gene therapy and provide the basis for future study and clinical practice.

Moreover, qRT-PCR analysis showed that the *eGFP* mRNA expression was the highest in cells transfected with CAG promoter, followed by cells transfected with mutant-404. These results are inconsistent with the results of protein expression levels, suggesting that the post-transcription process may affect transgene expression.

To further verify the effect of episomal vectors in primary cells, they were transfected into PEF cells. The results showed that the EF-1α promoter produced relatively higher eGFP expression compared with the CMV promoter in PEF cells. This finding may be because the CMV promoters used were unsuitable for primary cells, resulting in low transfection efficiency and low expression in primary cells. A previous study demonstrated that the promoter activity is dependent on the cell type [40]. Although CMV promoters are commonly used for the high expression of transgenes in mammalian cells [41,42], they may be inappropriate promoters for strong expression in primary cells. The EF-1α promoter can drive transgene expression in primary cells, but the expression level needs to be further improved. Future studies should attempt to improve the efficiency with other *cis*-acting elements, such as MARs and UCOEs.

In conclusion, the EF-1α promoter and mutant-404 provide higher enhancement of transient and stable transgene expression levels in two commonly used human cell lines, HEK293 and Chang liver cells. The mechanisms may be related to the type and quantity of TFREs. Moreover, qRT-PCR analysis showed that the mRNA expression levels are not related to protein expression levels in stably transfected mammalian cells. Furthermore, the EF-1α promoter conferred high transgene expression levels in primary cells, and the plasmid was also present in the episomal state. These results provided valuable information for improving transgene expression with episomal vectors in transfected mammalian cells. Therefore, we believe that this contribution is theoretically and practically valuable for gene therapy.

## Highlights

- EF-1α promoter and CMV promoter mutant greatly enhance transgene expression in HEK293 and Chang liver cells.
- In primary cells, High-level transgene expression was from EF-1α promoter.
- This study provided valuable information for transgene expression with episomal vector.
