# Supplementary material for: Effect of promoter, promoter mutation and enhancer on transgene expression mediated by episomal vectors in transfected HEK293, Chang liver and primary cells
Source: Bioengineered. 2019 Oct 31;10(1):548–60. doi: 10.1080/21655979.2019.1684863 (PMC6844389; doi:10.1080/21655979.2019.1684863)
Supplement: Supplemental Material [file kbie-10-01-1684863-s001.docx]

**Figure S1 Promoter and Enhancer sequences used in this study**

**CAG promoter sequence (1662bp)**

Tagttattaatagtaatcaattacggggtcattagttcatagcccatatatggagttccgcgttacataacttacggtaaatggcccgcctggctgaccgcccaacgacccccgcccattgacgtcaataatgacgtatgttcccatagtaacgccaatagggactttccattgacgtcaatgggtggagtatttacggtaaactgcccacttggcagtacatcaagtgtatcatatgccaagtacgccccctattgacgtcaatgacggtaaatggcccgcctggcattatgcccagtacatgaccttatgggactttcctacttggcagtacatctacgtattagtcatcgctattaccatggtcgaggtgagccccacgttctgcttcactctccccatctcccccccctccccacccccaattttgtatttatttattttttaattattttgtgcagcgatgggggcggggggggggggggggcgcgcgccaggcggggcggggcggggcgaggggcggggcggggcgaggcggagaggtgcggcggcagccaatcagagcggcgcgctccgaaagtttccttttatggcgaggcggcggcggcggcggccctataaaaagcgaagcgcgcggcgggcggggagtcgctgcgacgctgccttcgccccgtgccccgctccgccgccgcctcgcgccgcccgccccggctctgactgaccgcgttactcccacaggtgagcgggcgggacggcccttctcctccgggctgtaattagcgcttggtttaatgacggcttgtttcttttctgtggctgcgtgaaagccttgaggggctccgggagggccctttgtgcggggggagcggctcggggggtgcgtgcgtgtgtgtgtgcgtggggagcgccgcgtgcggctccgcgctgcccggcggctgtgagcgctgcgggcgcggcgcggggctttgtgcgctccgcagtgtgcgcgaggggagcgcggccgggggcggtgccccgcggtgcggggggggctgcgaggggaacaaaggctgcgtgcggggtgtgtgcgtgggggggtgagcagggggtgtgggcgcgtcggtcgggctgcaaccccccctgcacccccctccccgagttgctgagcacggcccggcttcgggtgcggggctccgtacggggcgtggcgcggggctcgccgtgccgggcggggggtggcggcaggtgggggtgccgggcggggcggggccgcctcgggccggggagggctcgggggaggggcgcggcggcccccggagcgccggcggctgtcgaggcgcggcgagccgcagccattgccttttatggtaatcgtgcgagagggcgcagggacttcctttgtcccaaatctgtgcggagccgaaatctgggaggcgccgccgcaccccctctagcgggcgcggggcgaagcggtgcggcgccggcaggaaggaaatgggcggggagggccttcgtgcgtcgccgcgccgccgtccccttctccctctccagcctcggggctgtccgcggggggacggctgccttcgggggggacggggcagggcggggttcggcttctggcgtgtgaccggcggctctagagcctctgctaaccatgttcatgccttcttctttttcctacag

**EF-1α promoter sequence (1335bp)**

GAGTAATTCATACAAAAGGACTCGCCCCTGCCTTGGGGAATCCCAGGGACCGTCGTTAAACTCCCACTAACGTAGAACCCAGAGATCGCTGCGTTCCCGCCCCCTCACCCGCCCGCTCTCGTCATCACTGAGGTGGAGAAGAGCATGCGTGAGGCTCCGGTGCCCGTCAGTGGGCAGAGCGCACATCGCCCACAGTCCCCGAGAAGTTGGGGGGAGGGGTCGGCAATTGAACCGGTGCCTAGAGAAGGTGGCGCGGGGTAAACTGGGAAAGTGATGTCGTGTACTGGCTCCGCCTTTTTCCCGAGGGTGGGGGAGAACCGTATATAAGTGCAGTAGTCGCCGTGAACGTTCTTTTTCGCAACGGGTTTGCCGCCAGAACACAGGTAAGTGCCGTGTGTGGTTCCCGCGGGCCTGGCCTCTTTACGGGTTATGGCCCTTGCGTGCCTTGAATTACTTCCACGCCCCTGGCTGCAGTACGTGATTCTTGATCCCGAGCTTCGGGTTGGAAGTGGGTGGGAGAGTTCGAGGCCTTGCGCTTAAGGAGCCCCTTCGCCTCGTGCTTGAGTTGAGGCCTGGCTTGGGCGCTGGGGCCGCCGCGTGCGAATCTGGTGGCACCTTCGCGCCTGTCTCGCTGCTTTCGATAAGTCTCTAGCCATTTAAAATTTTTGATGACCTGCTGCGACGCTTTTTTTCTGGCAAGATAGTCTTGTAAATGCGGGCCAAGATCTGCACACTGGTATTTCGGTTTTTGGGGCCGCGGGCGGCGACGGGGCCCGTGCGTCCCAGCGCACATGTTCGGCGAGGCGGGGCCTGCGAGCGCGGCCACCGAGAATCGGACGGGGGTAGTCTCAAGCTGGCCGGCCTGCTCTGGTGCCTGGCCTCGCGCCGCCGTGTATCGCCCCGCCCTGGGCGGCAAGGCTGGCCCGGTCGGCACCAGTTGCGTGAGCGGAAAGATGGCCGCTTCCCGGCCCTGCTGCAGGGAGCTCAAAATGGAGGACGCGGCGCTCGGGAGAGCGGGCGGGTGAGTCACCCACACAAAGGAAAAGGGCCTTTCCGTCCTCAGCCGTCGCTTCATGTGACTCCACGGAGTACCGGGCGCCGTCCAGGCACCTCGATTAGTTCTCGAGCTTTTGGAGTACGTCGTCTTTAGGTTGGGGGGAGGGGTTTTATGCGATGGAGTTTCCCCACACTGAGTGGGTGGAGACTGAAGTTAGGCCAGCTTGGCACTTGATGTAATTCTCCTTGGAATTTGCCCTTTTTGAGTTTGGATCTTGGTTCATTCTCAAGCCTCAGACAGTGGTTCAAAGTTTTTTTCTTCCATTTCAGGTGTCGTGA

**Enhancer 1 sequence (111 bp)**

**RE sequence** : NGEE′NE′E′NNNE′ENE′

**DNA sequence** : GGGACTTTCCGGGGCGGGGCACGTGGTGCACGGGACTTTC CGTGCACGTGCACGGGACTTTCCGGGACTTTCCGGGACTTTCCGTGCACCACGTGGGGACTTTCCGTGCAC

**Enhancer 2 sequence (117 bp)**

**RE sequence** :EEN′G′EN′E′N′N′E′N′N′N B

**DNA sequence**: CACGTGCACGTGCCTTTCAGGGGGGGCGGGGCACGTGCCT TTCAGGGGTGCACCCTTTCAGGGCCTTTCAGGGGTGCACCCTTTCAGGGCCTTTCAGGGGGGACTTTCCTTGCGCAA

**Enhancer 3 sequence (113 bp)**

**RE sequence** : N′NGNB′E′E′NBNE′NN′

**DNA sequence**:CCTTTCAGGGGGGACTTTCCGGGGCGGGGGGGACTTTCCAA CGCGTTGTGCACGTGCACGGGACTTTCCTTGCGCAAGGGACTTTCCGTGCACGGGACTTTCCCCTTTCAGGG

RE: regulatory element; N = NFκB, E = E-box, G = GC-box, B = C/EBPα ,**′** represent reverse orientation ( 3′ to 5′ ).

Nuclear factor kappa B (NFkB)=GGGACTTTCC;

CCAAT-enhancer binding protein alpha (C/EBPa)=TTGCGCAA;

GC-box =GGGGCGGGG;

Enhancer box (E-box)=CACGTG.
